# Risk of acute exacerbation of chronic obstructive pulmonary disease after COVID-19 recovery: a nationwide population-based cohort study

**DOI:** 10.1186/s12931-025-03123-x

**Published:** 2025-03-27

**Authors:** Sang Hyuk Kim, Hyun Lee, Min Ji Kim, Youlim Kim, Kyung Hoon Min, Kwang Ha Yoo, Jong Seung Kim, Ji-Yong Moon

**Affiliations:** 1 Division of Pulmonary, Allergy, and Critical Care Medicine, Department of Internal Medicine, Dongguk University Gyeongju Hospital, Dongguk University College of Medicine, Gyeongju, Republic of Korea; 2 Division of Pulmonary, Allergy, and Critical Care Medicine, Department of Internal Medicine, Korea University Guro Hospital, Korea University College of Medicine, Seoul, Republic of Korea; 3 Division of Pulmonary Medicine and Allergy, Department of Internal Medicine, Hanyang Medical Center, Hanyang University College of Medicine, Seoul, Republic of Korea; 4 Department of Medical Informatics, Jeonbuk National University Medical School, Jeonju, Republic of Korea; 5 Research Institute of Clinical Medicine of Jeonbuk National University, Biomedical Research Institute of Jeonbuk National University Hospital, Jeonju, Republic of Korea; 6 Department of Internal Medicine, Konkuk University Medical Center, Konkuk University School of Medicine, Seoul, Republic of Korea; 7 Department of Otorhinolaryngology-Head and Neck Surgery, Jeonbuk National University Medical School, Jeonju, Republic of Korea; 8 Division of Pulmonary, Allergy, and Critical Care Medicine, Department of Internal Medicine, Konkuk University Medical Center, Konkuk University School of Medicine, 120 Neungdong-ro, Gwangjin-gu, Seoul, 05030 Republic of Korea

**Keywords:** Lung Disease, Obstructive, Pulmonary Disease, Chronic Obstructive, Coronavirus, Exacerbation

## Abstract

**Background:**

Chronic obstructive pulmonary disease (COPD) is associated with severe Coronavirus disease 2019 (COVID-19) outcomes. However, it is uncertain whether the risk of acute exacerbation of COPD (AECOPD) increases after recovering from COVID-19.

**Methods:**

This study included 2,118 individuals with COPD from the Korea National Health Insurance Service database who were also diagnosed with COVID-19. Matched controls were chosen using 1:1 propensity score (PS) matching. We compared the risk of AECOPD after COVID-19 recovery between the COVID-19 cohort and matched controls between October 8, 2020, and December 31, 2021, using PS-matched Cox proportional hazard regression models.

**Results:**

During a median follow-up of 62 days (interquartile range, 29–179 days), including a median of 14 days of recovery time after COVID-19, 68 people (5.6%) in the COVID-19 cohort and 50 (3.9%) in the matched control group experienced AECOPD. Compared to the matched controls, the COVID-19 cohort had a significantly higher risk of overall AECOPD (hazard ratio [HR] = 1.45, 95% confidence interval [CI] = 1.09–1.92). This increased risk was particularly evident for severe AECOPD among individuals who had severe COVID-19 within the first 30days post-recovery (aHR = 8.14, 95% CI = 3.32–19.97). When classified by COVID-19 severity, while severe COVID-19 significantly increased this risk (aHR = 2.97, 95% CI = 2.15–4.11), non-severe COVID did not significantly influence the risk of AECOPD, regardless of time duration or exacerbation severity.

**Conclusion:**

Individuals with COPD who had severe COVID-19 have increased risk of AECOPD after COVID-19 recovery, especially within the first 30 days after COVID-19 recovery.

**Supplementary Information:**

The online version contains supplementary material available at 10.1186/s12931-025-03123-x.

## Introduction

The 2019 Coronavirus (COVID-19) pandemic has dramatically changed the global landscape of health management [1]. Although concerted global efforts to fight COVID-19 have led to the end of the pandemic, many countries still face the risk of COVID-19 outbreak [2]. Furthermore, in the endemic phase of COVID-19, concerns remain about the post-COVID-19 conditions of vulnerable populations [3].

Chronic obstructive pulmonary disease (COPD) is a major chronic respiratory disease characterized by respiratory symptoms and persistent airflow limitation [4]. This condition burdens individuals and healthcare systems significantly, with acute exacerbation of COPD (AECOPD) playing an important role in its severity and prognosis [5]. Despite advancements in COPD management, some individuals with COPD frequently experience AECOPD [6], which often leads to hospitalization, decreases in lung function, poor quality of life, and increased mortality [7]. The COVID-19 pandemic has further complicated this scenario, as individuals with COPD are at a higher risk of worse treatment outcomes from COVID-19 than are those without COPD [8]. However, most studies on COVID-19 and AECOPD have focused on decreased AECOPD rates during the pandemic period compared to those before the pandemic period due to the changes in the social environment (e.g., social distancing and mask use) [9–11]. Thus, it is largely unknown whether the risk of AECOPD increases after recovery from COVID-19. A recent prospective study performed in a single center showed that the risk of AECOPD increases after recovery from COVID-19 [12]. However, a single-center study design and relatively small number of participants limited the study findings in terms of generalizability. Thus, more evidence is needed to confirm the association between prior COVID-19 and the risk of AECOPD.

The Korea National Health Insurance System (NHIS) claims database has the advantage of enrolling a large number of representative samples from the Korean population. Using this advantage, we aimed to investigate the risk of AECOPD after COVID-19 recovery, with a focus on the time interval to exacerbation.

## Methods

### Data source

Our study used a retrospective cohort based on the NHIS claims database. This healthcare system, managed by the Korean government, provides universal health coverage to approximately 97% of the Korean population. The Korea NHIS provided demographic data (age, sex, resident area, income, and education status), anthropometric measurements (weight and height), and personal habits (smoking and alcohol drinking status) [13–16].

During the COVID-19 pandemic, the Korean government actively encouraged COVID-19 testing by providing financial assistance for COVID-19 diagnosis in all individuals exhibiting symptoms suggestive of SARS-CoV-2 viral infection. Additionally, throughout the COVID-19 pandemic, the Korean government offered financial support for the treatment of COVID-19 (NHIS-2022-1-623). The NHIS SARS-CoV-2 database contains testing results for all individuals who underwent SARS-CoV-2 testing. This database (*n* = 8,463,712) includes 561,009 individuals diagnosed with COVID-19 at least once between October 8, 2020, and December 31, 2021, and 7,902,703 individuals who were not diagnosed with COVID-19 during the same period. The data of COVID-19-naïve individuals were extracted from the NHIS database using randomized stratified sampling based on age and sex.

Several epidemiological studies have evaluated post-COVID-19 outcomes in individuals with chronic respiratory diseases using this database [17–19].

### Study population

From the NHIS SARS-CoV-2 database, 75,485 individuals met the COPD diagnosis criteria between October 7, 2010, and January 31, 2015. In this study, COPD was defined as at least two prescriptions for COPD-related drugs under the International Classification of Diseases (ICD-10) codes for COPD (J43.1, J43.2, J43.8, J43.9, or J44). COPD-related drugs included long-acting beta-2 agonist (LABA), long-acting muscarinic antagonist (LAMA), inhaled corticosteroids (ICS) plus LABA (ICS + LABA), LABA plus LAMA (LABA + LAMA), ICS plus LABA plus LAMA (ICS + LABA + LAMA), short-active muscarinic antagonists, short-acting beta-2 agonist, phosphodiesterase-4 inhibitor, systemic bronchodilators, and theophylline [20–22]. Of the initially enrolled individuals, those who did not undergo a health examination between 2019 and 2020 were excluded (*n* = 34,460). In the final COPD cohort, 41,025 individuals were included.

Among the COPD cohort, we further excluded individuals whose index date occurred after the last follow-up date, as well as those who had died before the index date. The index date was defined as the COVID-19 recovery date for the COVID-19 cohort and the corresponding date for matched controls. COVID-19 recovery was defined as two weeks from the COVID-19 diagnosis based on the observation that most patients recover within this timeframe [23]. However, for those hospitalized for more than 14 days after COVID-19 diagnosis, hospital discharge date was considered as COVID-19 recovery since studies evaluating long-term outcomes of hospitalized COVID-19 survivors followed the patients after hospital discharge [24, 25]. Accordingly, the COVID-19 recovery dates were (1) 14 days after COVID-19 diagnosis for non-hospitalized individuals, (2) 14 days after COVID-19 diagnosis for those hospitalized but discharged within this 14-day window period, and (3) hospital discharge date for those requiring hospitalization for more than 14 days after COVID-19 diagnosis. This process identified 2,118 individuals with COPD who had COVID-19 (COVID-19 cohort).

**Fig. 1:**
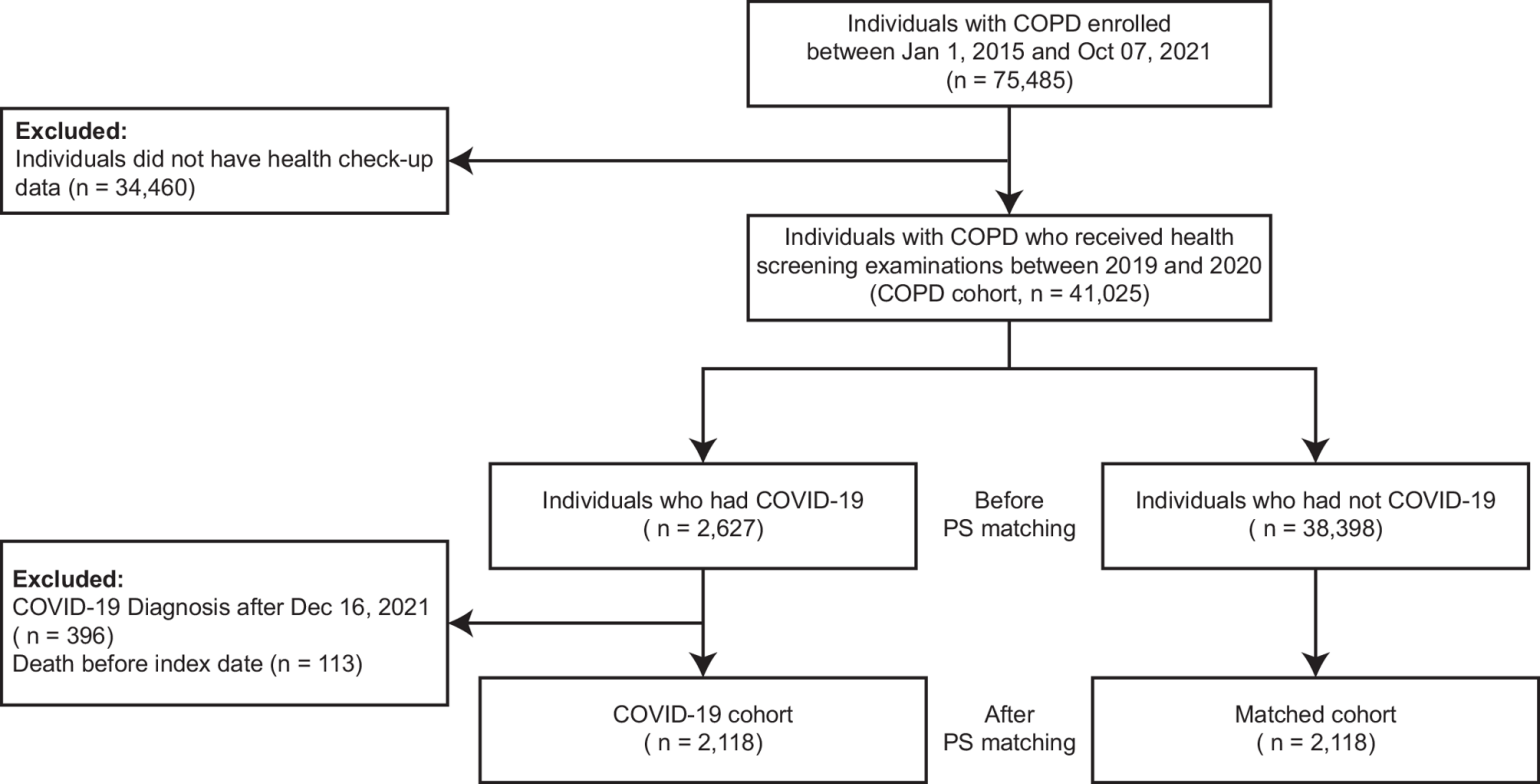
Flow chart of the study population ***Abbreviations***: COPD = chronic obstructive pulmonary disease, COVID-19 = Coronavirus disease 2019, PS = propensity score

Each individual in the COVID-19 cohort was matched to an individual with COPD who did not have COVID-19 based on age, sex, body mass index (BMI), smoking and alcohol drinking, income, resident area, and comorbidities (hypertension, diabetes, dyslipidemia, chronic kidney disease [CKD], and asthma) using 1:1 propensity score (PS) matching. The balance between these two groups was evaluated using the standard mean difference (SMD), with a value > 0.1 indicating an imbalance [26, 27]. After matching, 2,118 individuals with COPD who did not contract COVID-19 (matched controls) were included in the final analysis (Fig. 1).

### Exposure: COVID-19

COVID-19 was confirmed through positive results from real-time reverse transcription-polymerase chain reaction (RT-PCR) tests on nasal or pharyngeal swabs, with the ICD-10 code for COVID-19 (U07.1) [17, 28].

To consider COVID-19 severity, we further classified individuals who had COVID-19 into non-severe and severe cases. Individuals who required oxygen therapy, were admitted to the intensive care unit, or underwent mechanical ventilation or extracorporeal membrane oxygenation during COVID-19 treatment were defined as severe cases [17–19].

### Outcome: COPD exacerbation

The primary outcome was AECOPD [29], defined as (1) non-severe AECOPD: outpatient visits with ICD codes for COPD and concomitant prescription for systemic steroids or (2) severe AECOPD: either emergency room visits or hospitalization with ICD codes for COPD and concomitant prescription for systemic steroids [30, 31]. The enrolled individuals were followed from the index date to the occurrence of AECOPD, censoring events, or the final follow-up date (December 31, 2021), whichever came first.

### Covariates

BMI was calculated by dividing weight (kg) by height squared (m^2^) and categorized into five groups based on recommendations for the Asian population: underweight (< 18.5 kg/m^2^), normal (18.5–22.9 kg/m^2^), overweight (23.0–24.9 kg/m^2^), obese (25–29.9 kg/m^2^), and highly obese (≥ 30 kg/m^2^) [32]. Income level was classified into three groups: high (top 30%), low (bottom 30% and medical aid program recipients), and middle (the remaining). Regular physical activity was defined as either moderate physical activity for more than 30 min more than five days per week or vigorous physical activity for more than 20 min more than three days per week [33–35]. Residential regions were divided into metropolitan, mid-sized or small cities, and rural areas. Smoking and alcohol drinking were assessed using self-reported questionnaires. Smoking status was classified as never- or ever-smokers [36]. Alcohol drinking status was divided into none, 1–2 times weekly, 3–4 times weekly, and nearly every day. A history of severe AECOPD was defined as an emergency room visit or hospitalization with systemic steroid use under the ICD-10 codes for COPD within the previous year. Asthma was defined based on the ICD-10 codes J45–J46 and the prescription of asthma-related medications, including systemic steroids, bronchodilators, leukotriene receptor antagonists, and xanthine derivatives [17, 37–39]. Comorbidities were defined using ICD-10 codes as follows: hypertension (I10–I13 and I15), diabetes (E10–E14), dyslipidemia (E78), and CKD (N18) [15, 40–46].

### Statistical analysis

Categorical and continuous variables were presented as numbers and percentages and means and standard deviations (SDs). The χ^2^ and t-test were used for categorical and continuous variables, respectively, for comparisons between the COVID-19 cohort and matched controls. The incidence rate of AECOPD was determined by the ratio of AECOPD to the overall follow-up period, expressed in 10,000 person-years. Cumulative incidence curves were used to assess the difference in the incidence rate of AECOPD between the COVID-19 cohort and matched controls. The log-rank test was used to determine statistical significance.

We used PS-matched Cox proportional hazards regression for analyses comparing the COVID-19 cohort and the matched control group. In addition, to assess the time to exacerbation, the time intervals were divided into < 30 days, 30–90 days, and 90–180 days. For sensitivity analysis, we further performed the analysis using 2:1 PS matching. Statistical significance was determined by a two-sided *p*-value < 0.05. All statistical analyses were conducted using SAS version 9.4 (SAS Institute Inc., Cary, NC, USA) and R version 4.0.3 (R Foundation for Statistical Computing, Vienna, Austria).

## Results

### Baseline characteristics

As described in Table 1, no significant differences in baseline characteristics were observed between the COVID-19 cohorts and matched controls (all SMDs < 0.1).

**Table 1:** Baseline characteristics of the study population

	Matched cohort (*n* = 2,118)	COVID-19 cohort (*n* = 2,118)	SMD
**Age**, **years**, **mean (SD)**	68.3 (11.4)	68.7 (11.5)	0.03
**Age**, **years**			0.05
20–49	153 (7.2)	145 (6.8)	
50–59	200 (9.4)	218 (10.3)	
60–69	701 (33.1)	661 (31.2)	
70–79	746 (35.2)	760 (35.9)	
≥ 80	318 (15.0)	334 (15.8)	
**Sex**, **male**	1,441 (68.0)	1,416 (66.9)	0.03
**BMI**, **kg/m**^**2**^, **mean (SD)**	24.4 (3.69)	24.5 (3.54)	0.02
Low (< 18.5 kg/m^2^)	103 (4.9)	82 (3.9)	0.07
Normal (18.5–22.9 kg/m^2^)	619 (29.2)	625 (29.5)	
Overweight (23.0–24.9 kg/m^2^)	510 (24.1)	489 (23.1)	
Obese (25.0–29.9 kg/m^2^)	742 (35.0)	789 (37.3)	
Highly obese (≥ 30 kg/m^2^)	144 (6.8)	133 (6.3)	
**Regular physical activity**			< 0.01
No	1,546 (73.0)	1,542 (72.8)	
Yes	572 (27.0)	576 (27.2)	
**Smoking status**			0.01
Never smoker	1,068 (50.4)	1,082 (51.1)	
Past smoker	735 (34.7)	723 (34.1)	
Current smoker	315 (14.9)	313 (14.8)	
**Alcohol drinking status**			0.03
None	1,462 (69.0)	1,487 (70.2)	
1–2 times	386 (18.2)	367 (17.3)	
3–4 times	152 (7.2)	156 (7.4)	
Nearly every day	118 (5.6)	108 (5.1)	
**Income**			0.02
Low	516 (24.4)	527 (24.9)	
Middle	891 (42.1)	899 (42.4)	
High	711 (33.6)	692 (32.7)	
**Residential area**			0.02
Metropolitan cities	1,526 (72.0)	1,525 (72.0)	
Mid-size and small cities	432 (20.4)	423 (20.0)	
Rural areas	160 (7.6)	170 (8.0)	
**Severe AECOPD in the previous year**	257 (12.1)	258 (12.2)	< 0.01
**Comorbidities**			
Hypertension	903 (42.6)	925 (43.7)	0.02
Dyslipidemia	366 (17.3)	377 (17.8)	0.01
Diabetes mellitus	518 (24.5)	537 (25.4)	0.02
Chronic kidney disease	55 (2.6)	68 (3.2)	0.04
Asthma	805 (38.0)	869 (41.0)	0.06

Data are shown as number (%) or number (SD), as appropriate

***Abbreviations***: COVID-19 = coronavirus disease 2019, SMD = standard mean difference, SD = standard deviation, BMI = body mass index; AECOPD, acute exacerbation of chronic obstructive pulmonary disease

### Risk of AECOPD after recovery from COVID-19

The median follow-up duration was 62 days (interquartile range, 29–179 days), including a median of 14 days of recovery time after COVID-19. During the study period, 68 individuals (5.6%) in the COVID-19 cohort and 50 (3.9%) in the matched controls experienced AECOPD.

**Fig. 2:**
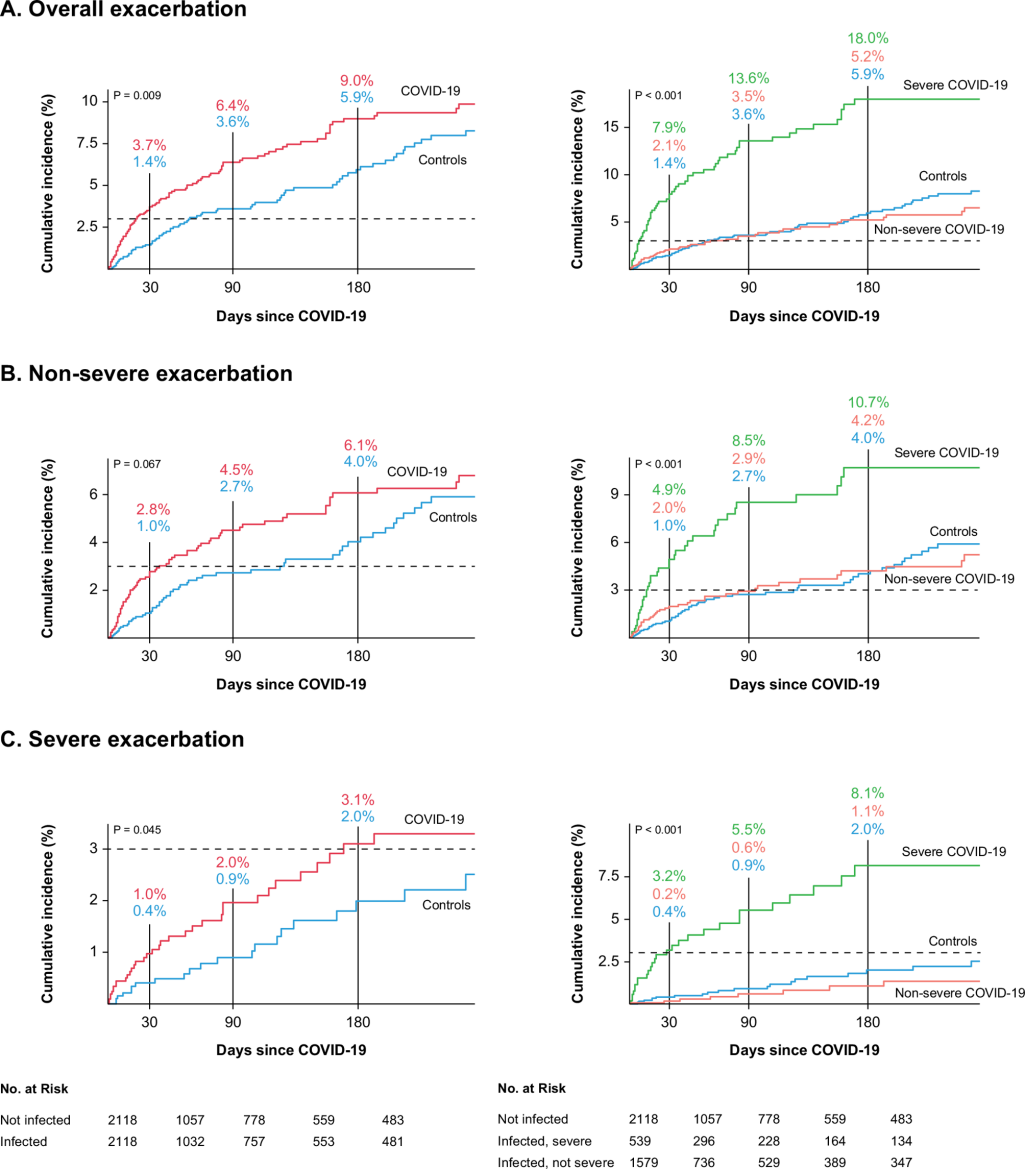
Cumulative incidence of AECOPD after COVID-19 recovery. The dashed lines represent a 3% cumulative incidence. The *p*-value was calculated using a log-rank test. (**A**) Overall exacerbation, (**B**) non-severe AECOPD, and (**C**) severe AECOPD

Figure 2A demonstrated this difference using cumulative incidence plots (log-rank *p* = 0.009). As shown in Table 2, there was a significant increase in the risk of AECOPD after COVID-19 recovery in the COVID-19 cohort compared to that in the matched controls (hazard ratio [HR] = 1.46, 95% confidence interval [CI] = 1.10–1.93). In terms of exacerbation severity, COVID-19 was associated with an increased risk of severe AECOPD (HR = 1.70, 95% CI = 1.01–2.89), but it did not significantly increase the risk of non-severe AECOPD (HR = 1.36, 95% CI = 0.98–1.91). Similar results were found in the cumulative incidence plots (Fig. 2B, log-rank *p* = 0.067; Fig. 2C, log-rank *p* = 0.045). Conversely, no significant association was identified between non-severe COVID-19 and the risk of AECOPD.

**Table 2:** Risk of AECOPD after COVID-19 recovery in individuals with COPD

Outcome	Groups	*N* at risk	AECOPD (*n*)	AECOPD rate (/10,000 PY)	PS-matched Cox results; HR (95% CI)
Overall AECOPD	**Matched controls**	2,118	82	1328.75	Reference
	**COVID-19 cohort**	2,118	118	1926.48	1.46 (1.10–1.93)
	With non-severe COVID-19	1,579	51	1164.56	0.87 (0.61–1.24)
	With severe COVID-19	539	67	3837.76	2.97 (2.15–4.11)
Non-severe AECOPD	**Matched controls**	2,118	60	972.25	Reference
	**COVID-19 cohort**	2,118	81	1322.42	1.36 (0.98–1.91)
	With non-severe COVID-19	1,579	43	981.88	0.51 (0.23–1.15)
	With severe COVID-19	539	38	2176.64	4.77 (2.74–8.30)
Severe AECOPD	**Matched controls**	2,118	22	356.49	Reference
	**COVID-19 cohort**	2,118	37	604.07	1.70 (1.01–2.89)
	With non-severe COVID-19	1,579	8	182.68	1.00 (0.68–1.48)
	With severe COVID-19	539	29	1661.12	2.31 (1.54–3.47)

Data are shown as number or ratio (95% CI), as appropriate

***Abbreviations***: AECOPD = acute exacerbation of chronic obstructive pulmonary disease; COVID-19 = Coronavirus disease 2019, COPD = chronic obstructive pulmonary disease, HR = hazard ratio, CI = confidence interval

### Stratified analysis according to COVID-19 severity and time interval

As shown in Table 3, within the first 30 days after COVID-19 recovery, individuals who had severe COVID-19 had a pronounced risk of overall AECOPD compared to that of the matched controls (HR = 5.77, 95% CI = 3.48–9.55; Fig. 2A, log-rank test *p* < 0.001). The increased risk was observed not only for non-severe AECOPD (HR = 4.84, 95% CI = 2.61–8.97) but also for severe AECOPD (HR = 8.14, 95% CI = 3.32–19.97) (Fig. 2B and C, respectively, log-rank test *p* < 0.001 for both).

**Table 3:** Time-dependent analysis for the risk of AECOPD after COVID-19 recovery

Outcome	Groups	PS-matched Cox results; HR (95% CI)		
		< 30 days	30–90 days	90–180 days
Overall AECOPD	**Matched controls**	Ref.	Ref.	Ref.
	**COVID-19 cohort**			
	Total	**2.63 (1.66–4.17)**	1.19 (0.69–2.06)	0.85 (0.48–1.51)
	With non-severe COVID-19	1.49 (0.86–2.57)	0.60 (0.28–1.24)	0.71 (0.37–1.39)
	With severe COVID-19	**5.77 (3.48–9.55)**	**2.70 (1.47–4.98)**	1.20 (0.56–2.56)
Non-severe AECOPD	**Matched controls**	Ref.	Ref.	Ref.
	**COVID-19 cohort**			
	Total	**2.70 (1.57–4.64)**	0.97 (0.51–1.85)	0.83 (0.41–1.68)
	With non-severe COVID-19	1.92 (1.05–3.51)	0.53 (0.22–1.25)	0.83 (0.38–1.82)
	With severe COVID-19	**4.84 (2.61–8.97)**	2.09 (0.99–4.38)	0.82 (0.28–2.43)
Severe AECOPD	**Matched controls**	Ref.	Ref.	Ref.
	**COVID-19 cohort**			
	Total	**2.45 (1.02–5.92)**	2.05 (0.70–5.98)	0.90 (0.35–2.34)
	With non-severe COVID-19	0.39 (0.08–1.89)	0.86 (0.21–3.59)	0.48 (0.13–1.78)
	With severe COVID-19	**8.14 (3.32–19.97)**	**5.02 (1.59–15.83)**	1.90 (0.64–5.67)

Data are shown as number or ratio (95% CI), as appropriate

Abbreviations: AECOPD = acute exacerbation of chronic obstructive pulmonary disease; COVID-19 = Coronavirus disease 2019, HR = hazard ratio, CI = confidence interval

During the 30–90 days after COVID-19 recovery, the risk of overall and severe AECOPD remained significantly increased in individuals who had severe COVID-19 compared to those in the matched controls (HR for overall AECOPD = 2.70, 95% CI = 1.47–4.98; HR for severe AECOPD = 5.02, 95% CI = 1.59–15.83). The risks of overall, non-severe, and severe AECOPD were diminished by 90 days in the COVID-19 cohort compared to the controls, even among severe COVID-19 cases. Meanwhile, non-severe COVID-19 did not increase the risk of AECOPD, regardless of time duration and exacerbation severity.

### Sensitivity analysis

As shown in Table S1 and Figure S1, the sensitivity analysis results based on 2:1 PS matching showed similar results.

## Discussion

In this study, the risk of AECOPD after COVID-19 recovery was significantly higher in the COVID-19 cohort than in matched controls. The detailed significant findings from our nationwide large-scale study are as follows. First, while following individuals with COPD for up to 180 days after COVID-19, 5.6% in the COVID-19 cohort experienced AECOPD, while 3.9% of matched controls experienced AECOPD. Second, the increased risk of AECOPD was only pronounced in individuals who had severe COVID-19. Third, these increased risks of AECOPD were highest within the first 30 days and diminished by 90 days after COVID-19 recovery.

There has been limited information on the risk of AECOPD after recovery from COVID-19 in individuals with COPD. While Hyams and colleagues thoroughly explored the direct effect of COVID-19 on AECOPD [47], they did not evaluate the risk of AECOPD after COVID-19 recovery. To the best of our knowledge, only one single-center observational study evaluated the risk of AECOPD after COVID-19 [12]. In that study, compared to those who did not have COVID-19, individuals with COPD who had severe COVID-19 had 4.7-fold higher odds of experiencing severe AECOPD; in contrast, those who had non-severe COVID-19 did not have an increased risk of AECOPD. However, the relatively small number of individuals who had severe COVID-19 (*n* = 37) in a single center requires further evaluation using a larger population. The use of nationwide representative data could have allowed confirmation of previous findings, providing solid evidence on the risk of AECOPD after COVID-19 recovery. Furthermore, a comprehensive analysis performed after matching the possible confounders strengthens the robustness of our results.

It remains unclear whether different causes of exacerbation lead to distinct clinical courses, particularly regarding recurrent exacerbations. A recent systematic review and meta-analysis reported 30-, 60-, and 90-day readmission rates for AECOPD as 11%, 17%, and 17%, respectively [48]. These rates are higher than those observed in our study, likely because the studies in the meta-analysis were based on hospitalized patients. Additionally, factors such as sex, number of hospitalizations, length of stay, and certain comorbidities, including heart failure or cancer, were identified as risk factors. As demonstrated in this study, multiple factors influence the risk of recurrent AECOPD, making it insufficient to predict outcomes based solely on exacerbation causes. Nevertheless, the underlying causes of exacerbation may significantly impact AECOPD management through the lens of the etioendophenotype [49]. Unfortunately, our study could not assess whether COVID-19-related exacerbations pose a higher risk than other types of exacerbations, as specific causes of exacerbation could not be provided. Future studies focusing on the causes of exacerbations would provide valuable insights.

Beyond the replication of the previous findings, our study has novelty in terms of incorporating time-dependent analysis when exploring the association between AECOPD and COVID-19, particularly in relation to COVID-19 severity. This complex analysis revealed two important findings. First, the major contributor of AECOPD after COVID-19 occurred in individuals who had severe COVID-19. Second, while the increased risk of exacerbation persisted for the first month after COVID-19 recovery in cases of non-severe AECOPD, the increased risk of severe AECOPD in individuals who had experienced severe COVID-19 persisted longer, i.e., during the first three months after COVID-19 recovery.

Another notable finding of this study is that non-severe COVID-19 was not associated with an increased risk of AECOPD. The varying clinical spectrum of COVID-19, ranging from asymptomatic carriers to respiratory failure, has been well-documented [50]. Therefore, clinicians should prioritize the severity of COVID-19 over the mere presence of infection. For COPD patients with mild COVID-19, a similar approach to managing other high-risk populations, such as antiviral therapy and quarantine recommendations, may suffice [51]. However, as our study did not fully address the impact of COPD severity, a more cautious approach might be necessary for patients with severe COPD.

Our findings necessitate an urgent reassessment of preventive strategies to reduce the risk of AECOPD in individuals with COPD after COVID-19 recovery, especially in individuals who experienced severe COVID-19. Individuals discharged from hospitals after severe COVID-19 warrant more intensive observation within the first 90 days after recovery to detect and promptly address any signs of AECOPD. Considering the novel challenges posed by the post-COVID-19 environment, this issue should be discussed actively, including in future COPD guidelines.

We should acknowledge the limitations of this study. First, our study enrolled individuals who participated in health screening examinations. Thus, a healthy user bias should be considered when interpreting our results. Second, we defined COPD and other comorbidities using ICD-10 codes. There might have resulted in inaccurate diagnosis, which is an inherent limitation of studies using a claim-based database. Third, other confounding factors, such as pulmonary function and COPD subtypes, were not considered due to unavailability of this information in the NHIS database. Additionally, we could not account for differences in the level of care between the COVID-19 cohort and controls. Therefore, future studies evaluating the association between COVID-19 and the risk of AECOPD should consider this issue. Fourth, other viral infections may have occurred during the follow-up period, potentially contributing to AECOPD. However, considering the widespread implementation of social and personal protective measures during the pandemic and several studies reporting a reduced overall exacerbation rate during this time [52], the impact of this factor is likely minimal [53, 54]. Finally, our findings were obtained from Korea, which may limit their generalizability. For example, BMI categories were determined based on Asian BMI guidelines, which do not match those used in other parts of the world.

In conclusion, individuals with COPD have an increased risk of AECOPD after COVID-19 recovery. This finding is more evident in severe COVID-19 cases within the first 30 days after COVID-19 recovery. In addition, the increased risk of severe AECOPD in individuals who experienced severe COVID-19 persisted for 90 days after COVID-19 recovery.

## Electronic supplementary material

Below is the link to the electronic supplementary material.

Supplementary Table S1: Risk of AECOPD after COVID-19 recovery in individuals with COPD after 2:1 propensity score matching

Supplementary Figure S1: Cumulative incidence of AECOPD after COVID-19 recovery after 2:1 propensity score matching. The dashed lines represent a 3% cumulative incidence. The *p*-value was calculated using a log-rank test. (**A**) Overall exacerbation, (**B**) non-severe AECOPD, and (**C**) severe AECOPD. Abbreviations: AECOPD = acute exacerbation of chronic obstructive pulmonary disease; COVID-19 = Coronavirus disease 2019

## Data Availability

The data that support the findings of this study are available from the Korea NHIS but restrictions apply to the availability of these data, which were used under license for the current study, and so are not publicly available. Data are however available from the authors upon reasonable request and with permission of the Korea NHIS.

## Abbreviations

AECOPD: Acute Exacerbation of Chronic Obstructive Pulmonary Disease
BMI: Body Mass Index
CKD: Chronic Kidney Disease
COPD: Chronic Obstructive Pulmonary Disease
CI: Confidence Interval
COVID-19: Coronavirus Disease 2019
ICS: Inhaled Corticosteroids
ICD-10: International Classification of Diseases, 10th Revision
LABA: Long-Acting Beta-2 Agonist
LAMA: Long-Acting Muscarinic Antagonist
NHIS: National Health Insurance System
PS: Propensity Score
RT-PCR: Real-Time Reverse Transcription-Polymerase Chain Reaction
SMD: Standard Mean Difference
SARS-CoV-2: Severe Acute Respiratory Syndrome Coronavirus 2
SD: Standard Deviation
aHR: Adjusted Hazard Ratio
