# Supplementary material for: Risk of acute exacerbation of chronic obstructive pulmonary disease after COVID-19 recovery: a nationwide population-based cohort study
Source: Respir Res. 2025 Mar 27;26:116. doi: 10.1186/s12931-025-03123-x (PMC11951598; doi:10.1186/s12931-025-03123-x)
Supplement: Supplementary file 1 — Supplementary Table S1: Risk of AECOPD after COVID-19 recovery in individuals with COPD after 2:1 propensity score matching [file 12931_2025_3123_MOESM1_ESM.docx]

***Table S1:* Risk of AECOPD after COVID-19 recovery in individuals with COPD after 2:1 propensity score matching**

| **Outcome** | **Groups** | **N at risk** | **AECOPD**  **(n)** | **AECOPD rate**  **(/10,000 PY)** | **PS-matched Cox results; HR**  **(95% CI)** | **Multivariable Cox results; HR (95% CI)** |
| --- | --- | --- | --- | --- | --- | --- |
| Overall AECOPD | **Matched controls** | 4,236 | 157 | 1401.57 | Reference | Reference |
|  | **COVID-19 cohort** | 2,118 | 115 | 2076.39 | 1.48 (1.17–1.89) | N/A |
|  | With non-severe COVID-19 | 1,579 | 50 | 1260.55 | N/A | 0.96 (0.70–1.32) |
|  | With severe COVID-19 | 539 | 65 | 4135.00 | N/A | 3.02 (2.26–4.03) |
| Non-severe AECOPD | **Matched controls** | 4,236 | 115 | 1026.63 | Reference | Reference |
|  | **COVID-19 cohort** | 2,118 | 80 | 1444.44 | 1.41 (1.06–1.87) | N/A |
|  | With non-severe COVID-19 | 1,579 | 42 | 1058.86 | N/A | 1.06 (0.74–1.51) |
|  | With severe COVID-19 | 539 | 38 | 2417.39 | N/A | 2.18 (1.50–3.16) |
| Severe AECOPD | **Matched controls** | 4,236 | 42 | 374.94 | Reference | Reference |
|  | **COVID-19 cohort** | 2,118 | 35 | 631.94 | 1.69 (1.08–2.64) | N/A |
|  | With non-severe COVID-19 | 1,579 | 8 | 201.69 | N/A | 0.64 (0.30–1.36) |
|  | With severe COVID-19 | 539 | 27 | 1717.62 | N/A | 3.39 (2.07–5.54) |

Data are shown as number or ratio (95% CI), as appropriate.

***Abbreviations***: AECOPD = acute exacerbation of chronic obstructive pulmonary disease; COVID-19 = Coronavirus disease 2019, COPD = chronic obstructive pulmonary disease, HR = hazard ratio, CI = confidence interval.
