# Supplementary figures and images for: Risk of acute exacerbation of chronic obstructive pulmonary disease after COVID-19 recovery: a nationwide population-based cohort study
Source: Respir Res. 2025 Mar 27;26:116. doi: 10.1186/s12931-025-03123-x (PMC11951598; doi:10.1186/s12931-025-03123-x)

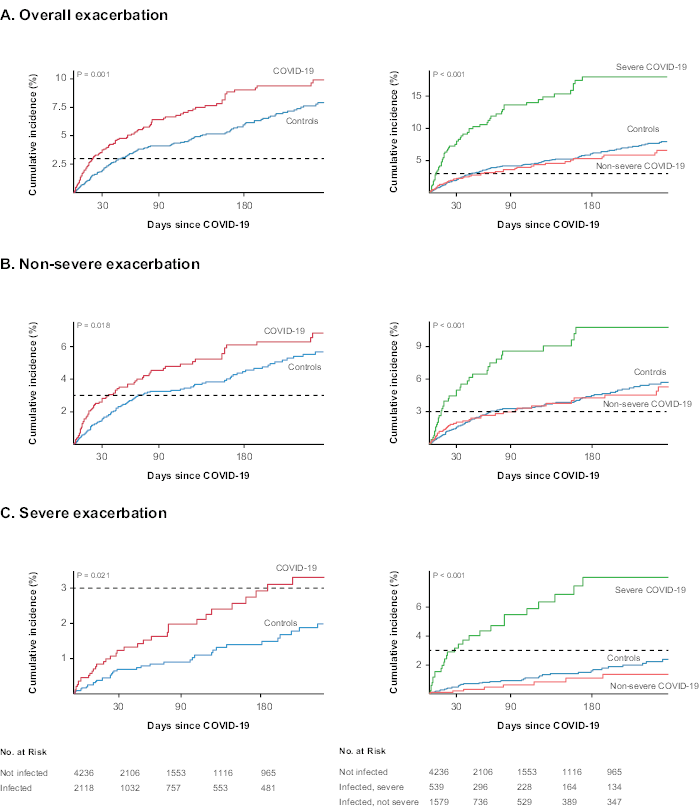

Supplement: Supplementary file 2 — Supplementary Figure S1: Cumulative incidence of AECOPD after COVID-19 recovery after 2:1 propensity score matching. The dashed lines represent a 3% cumulative incidence. The p-value was calculated using a log-rank test. (A) Overall exacerbation, (B) non-severe AECOPD, and (C) severe AECOPD. Abbreviations: AECOPD = acute exacerbation of chronic obstructive pulmonary disease; COVID-19 = Coronavirus disease 2019 [file 12931_2025_3123_MOESM2_ESM.png]
